# Ameliorated Autoimmune Arthritis and Impaired B Cell Receptor-Mediated Ca^2+^ Influx in Nkx2-3 Knock-out Mice

**DOI:** 10.3390/ijms21176162

**Published:** 2020-08-26

**Authors:** Esam Khanfar, Katalin Olasz, Fanni Gábris, Erzsébet Gajdócsi, Bálint Botz, Tamás Kiss, Réka Kugyelka, Tímea Berki, Péter Balogh, Ferenc Boldizsár

**Affiliations:** 1 Department of Immunology and Biotechnology, Medical School, University of Pécs, 7624 Pécs, Hungary; esam.khanfar@pte.hu (E.K.); olasz.katalin@pte.hu (K.O.); gabris.fanni@pte.hu (F.G.); gajdocsi.erzsebet@pte.hu (E.G.); reka.kugyelka@gmail.com (R.K.); berki.timea@pte.hu (T.B.); balogh.peter@pte.hu (P.B.); 2 Lymphoid Organogenesis Research Group, János Szentágothai Research Centre, University of Pécs, 7624 Pécs, Hungary; 3 Department of Medical Imaging, Medical School, University of Pécs, 7624 Pécs, Hungary; balint.botz@gmail.com; 4 Molecular Pharmacology Research Group, János Szentágothai Research Centre and Centre for Neuroscience, University of Pécs, 7624 Pécs, Hungary; kiss891012@gmail.com; 5 Department of Pharmacology and Pharmacotherapy, Medical School, University of Pécs, 7624 Pécs, Hungary

**Keywords:** autoimmune arthritis, Nkx2-3, B cell activation

## Abstract

B cells play a crucial role in the pathogenesis of rheumatoid arthritis. In Nkx2-3-deficient mice (Nkx2-3^−/−^) the spleen’s histological structure is fundamentally changed; therefore, B cell homeostasis is seriously disturbed. Based on this, we were curious, whether autoimmune arthritis could be induced in Nkx2-3^−/−^ mice and how B cell activation and function were affected. We induced arthritis with immunization of recombinant human proteoglycan aggrecan G1 domain in Nkx2-3^−/−^ and control BALB/c mice. We followed the clinical picture, characterized the radiological changes, the immune response, and intracellular Ca^2+^ signaling of B cells. Incidence of the autoimmune arthritis was lower, and the disease severity was milder in Nkx2-3^−/−^ mice than in control BALB/c mice. The radiological changes were in line with the clinical picture. In Nkx2-3^−/−^ mice, we measured decreased antigen-induced proliferation and cytokine production in spleen cell cultures; in the sera, we found less anti-CCP-IgG2a, IL-17 and IFNγ, but more IL-1β, IL-4 and IL-6. B cells isolated from the lymph nodes of Nkx2-3^−/−^ mice showed decreased intracellular Ca^2+^ signaling compared to those isolated from BALB/c mice. Our findings show that the transcription factor Nkx2-3 might regulate the development of autoimmune arthritis most likely through modifying B cell activation.

## 1. Introduction

Rheumatoid arthritis (RA) is the most frequent systemic autoimmune disease in the Western-European and North-American countries [1]. The disease primarily affects the small joints, where a chronic, progressive inflammation leads to cartilage and bone destruction, associated with severe pain and disability [1]. Despite intensive research, no definitive cause(s) of the disease are known, and therefore, unfortunately, no curative treatment is available to date [2]. So, finding the potential pathogenic factors and mechanisms in the background of RA is of utmost importance because they might provide a basis for future therapies. In this regard, mouse models of RA are extremely useful because several aspects of the disease can be studied more efficiently than in humans [3]. Especially those models are beneficial, which share many features of RA, like proteoglycan-aggrecan-induced arthritis (PGIA) [4] and its refined version, recombinant human G1 domain-induced arthritis (GIA) [5]. (P)GIA is similar to RA in many respect: (i) clinical picture [4,5], (ii) histological changes [4,5], (iii) radiological changes [4,6], (iv) autoreactive T cell activation [7], (v) Th1 and Th17 differentiation [8], (vi) production of autoantibodies (both against the mouse cartilage aggrecan and citrullinated antigens) [5] and (vii) proinflammatory cytokines was described [5].

RA is a chronic, progressive disease, usually lasting for decades [2,9,10]. According to our present view on RA pathogenesis, the patients are diagnosed only in the final inflammatory/destructive phase of the disease, when the typical symptoms (pain, swollen joints) appear [10]. However, the loss of tolerance and the development of symptomless autoimmunity precedes this usually by several years [10]. The dysregulation of the immune system might be the result of the interplay between genetic (*MHC* and *non-MHC* genes), epigenetic and environmental factors (infections, diet, smoking) [9,10]. The development of autoreactive T cells and starting of autoantibody production might be the key elements of this preclinical/latent phase of RA [10] and likewise, during the initiation period of its model PGIA [8]. These processes most likely take place not only locally, in the joints, but, importantly, in the lymphatic tissues like the lymph nodes and the spleen [2,8].

The spleen is well known for its function in the degradation of red blood cells and the immune response against blood-borne antigens, especially those of encapsulated bacteria [11,12]. Moreover, the spleen is critical for B cell development and, uniquely, all major peripheral B cell populations (B1a-, B1b-, B2-, marginal zone (MZ) B cells) can be found here. Not much is known about the exact role of the spleen in RA, however, based on mouse models of GIA and collagen-induced arthritis (CIA), we might suspect a potential involvement: increased size and more activated cells can be detected in spleens from both GIA (own unpublished observation) or CIA mice [13].

Nirenberg-Kim (NK) 2 homeobox 3 (Nkx2-3) is a homeodomain transcription factor, which is essential for the normal development of the spleen, Peyer’s patches and small intestine [14,15,16,17]. Along with its role in the development of intestinal lymphoid tissues. Nkx2-3 is crucial for the expression and regulation of the mucosal addressin cell adhesion molecule-1 (MADCAM-1) on the spleen sinus lining cells and on high endothelial venules of the mesenteric lymph nodes and Peyer’s patches [17,18,19,20]. Nkx2-3 has an important role in spleen organization and function, since it controls the correct micro-environment for B cell maturation and T-cell-dependent (TD) immune reaction [14,21]. Its absence results in disorganized germinal center (GC) formation leading to abnormal secondary B cell differentiation and decreased antibody response with minimal affinity maturation [21,22]. Nkx2-3-deficient mice (Nkx2-3^−/−^) are either asplenic or have a significantly reduced spleen size with a lack of the marginal zone [21]. In response to the TD antigen, the number of circulating lymphocytes of the Nkx2-3^−/−^ mice was found to be increased compared to both Nkx2-3^+/−^ and Nkx2-3^+/+^ [21] indicating their altered distribution between peripheral lymphoid tissues. Moreover, Nkx2-3^−/−^ mice showed an elevation in the number of the B cells in mesenteric lymph nodes which may be due to the abnormal development of the small intestine and the Peyer’s patches of these mice, and also a significant increase in the number of the IgM^+^ B cells in the bone marrow (BM) [21].

In humans, overexpression of Nkx2-3 was found to be associated with both Crohn’s disease and ulcerative colitis through its effect on the regulation of PTPN2 expression, VEGF and MADCAM-1 signaling, and the production of endothelin-1 [16,18,23,24]. Additionally, Robles and colleagues reported that chromosomal translocation of *Nkx2-3* gene alongside with immunoglobulin heavy chain gene (*IGH*), resulted in irregular B cell receptor signaling leading to the MZ B cell lymphomagenesis, through the activation of the NF-KB and PI3K-AKT pathways [25].

Our aim in this study was to investigate the effect of Nkx2-3 deficiency in GIA, a mouse model of autoimmune arthritis, and study the effect of Nkx2-3 absence on B cell signaling and activation. Here, we report for the first time that GIA can be induced in Nkx2-3^−/−^ mice, although with lower incidence, decreased severity and less joint destruction. We measured decreased T cell proliferation and cytokine production in spleen cultures. We found less anti-CCP-IgG2a, IL-17 and IFNγ, but more IL-1β, IL-4 and IL-6 in the sera. Finally, B cells of Nkx2-3^−/−^ mice showed decreased intracellular Ca^2+^ signaling compared to those isolated from BALB/c mice. Collectively, these data indicate that Nkx2-3 mice are relatively resistant to GIA-induction which correlates with their impaired in vitro B cell responsiveness.

## 2. Results

### 2.1. Decreased Severity and Incidence of rhG1-Induced Arthritis in Nkx2-3 Knock-Out Mice

The spleen plays a critical role in the correct development and recirculation of B cell populations. The connection between the peritoneal B cell pool and the splenic B cells is also well established [26]. Recombinant human G1-induced arthritis is provoked by repeated intraperitoneal injections of the aggrecan G1 domain in the dimethyl-dioctadecyl-ammonium (DDA) adjuvant. By intraperitoneal immunization the first antigen encounter occurs in the peritoneal cavity, but soon the antigen is transported to other lymphoid tissues like the local lymph nodes through lymph vessels and the spleen through blood vessels. In GIA, both the local peritoneal activation of immune cells and the activation of the splenic cells are thought to be critical for the development of autoimmune arthritis [8].

**Figure 1:**
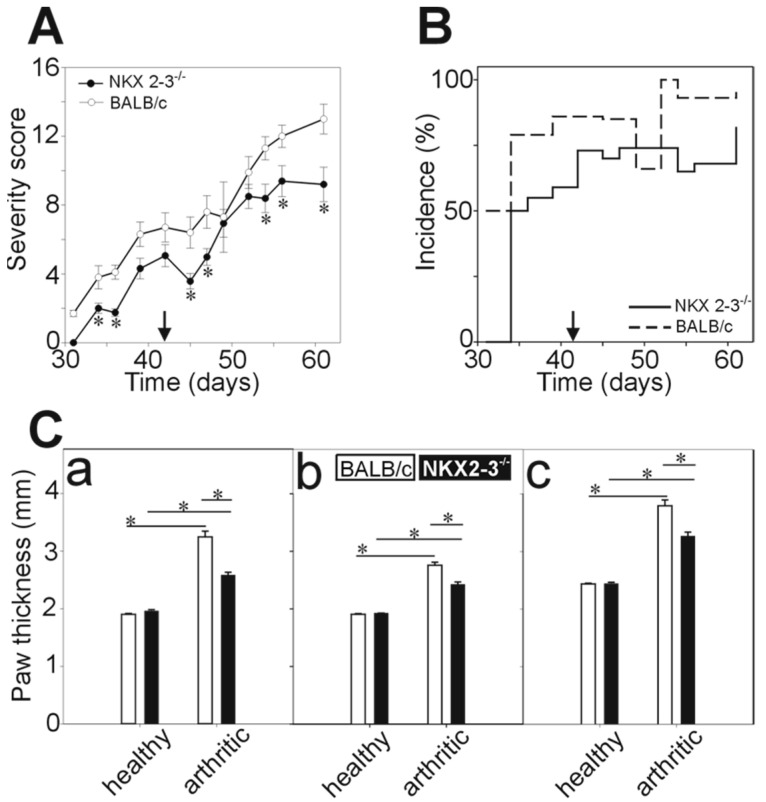
The comparison of the clinical parameters of recombinant human G1 (rhG1)-induced arthritis (GIA) in Nkx2-3^−/−^ and control BALB/c mice. Female Nkx2-3^−/−^ (*n* = 40) and control BALB/c (*n* = 27) mice were immunized with rhG1 and dimethyl-dioctadecyl-ammonium (DDA) adjuvant intraperitoneally three times every third week. The severity score (**A**) and incidence (**B**) of the induced arthritis is shown on the diagrams. Black arrows show the time of the third immunization (Day 42). Severity of the disease was determined every second day with the help of a scoring system ranging from 1 to 4, based on the swelling, redness and ankylosis of the joints of the paws. Clinical scores are visualized as mean ± standard error of mean (SEM). The thickness of the limbs (**C**) were measured with a digital caliper two weeks after the third immunization. The diagrams show the thickness values of the wrist (**C/a**), legs (**C/b**) and ankles (**C/c**) as mean ± SEM. Statistically significant differences (∗ *p* < 0.05) are indicated.

Since Nkx2-3 knock-out mice were present with severe splenic developmental defects, we were curious to test whether rhG1-induced arthritis could be observed in them. To this end, we immunized Nkx2-3^−/−^ and wild-type control BALB/c mice side-by-side. Nkx2-3^−/−^ mice developed arthritis; however, to a lesser extent than the control BALB/c mice indicated by both the lower clinical severity scores (9.2 ± 1.0 in Nkx2-3^−/−^ versus 13.0 ± 0.9 in BALB/c controls at Day 61) (Figure 1A) and the lower incidence (~70% in Nkx2-3^−/−^ versus >90% in BALB/c controls after the third immunization) (Figure 1B). The clinical scores were supported by the limb thickness measurements, too. We measured the wrist (Figure 1(Ca)), leg (Figure 1(Cb)) and ankle (Figure 1(Cc)) thickness two weeks after the third immunization. We found that in arthritic Nkx2-3^−/−^ mice, the limbs were significantly less swollen (Figure 1(Ca–Cc)) indicating a lower degree of inflammatory edema. Interestingly, in Nkx2-3^−/−^ mice, the disease progression was also different from that seen in BALB/c mice: in GIA, typically, the arthritis develops progressively and once established it will not regress, however, in the case of Nkx2-3^−/−^ mice we have seen the undulation of paw inflammation in some cases.

### 2.2. Micro-CT Imaging Confirmed Decreased Cartilage and Bone Destruction in Arthritic Nkx2-3 Knock-Out Mice

**Figure 2:**
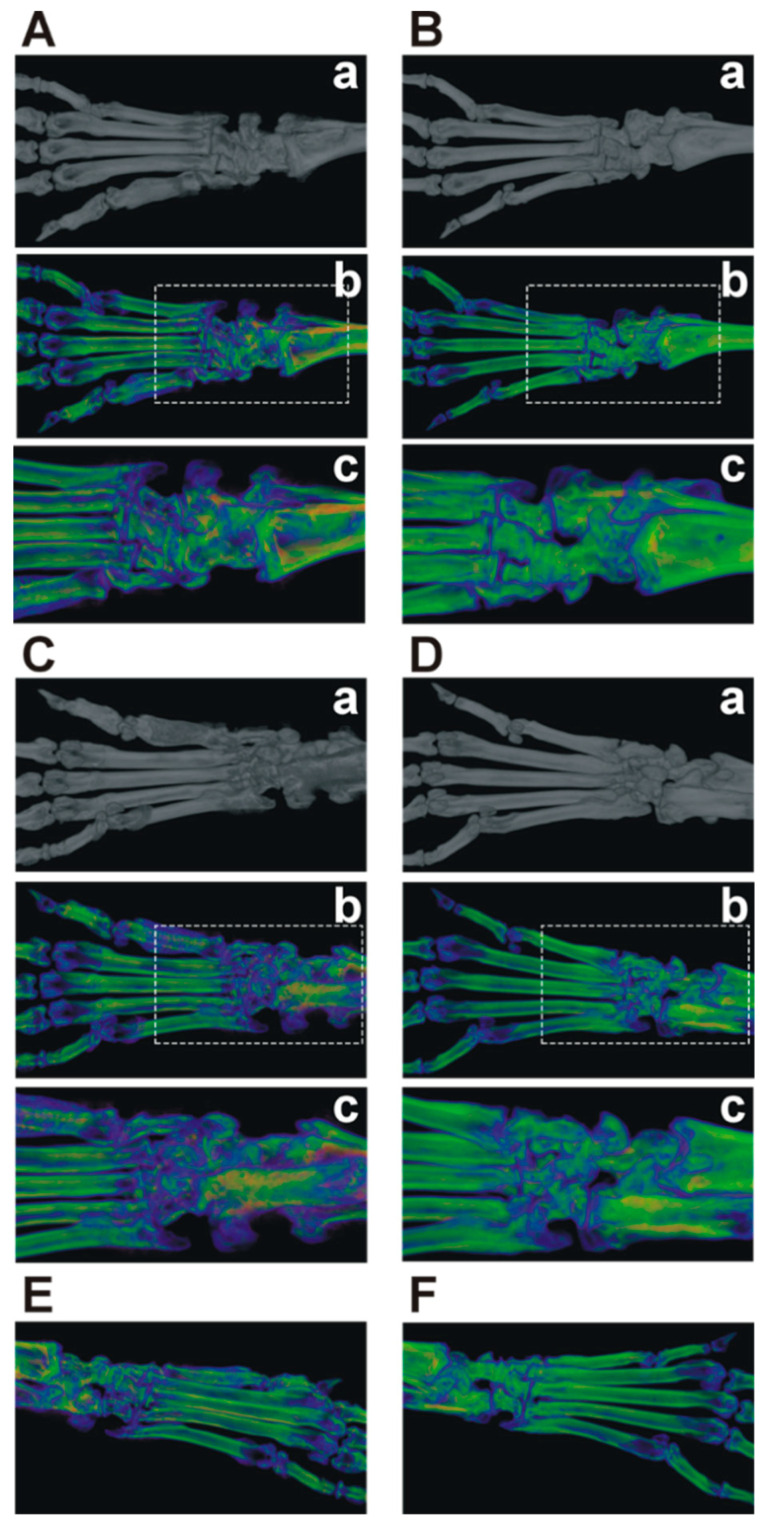
The comparison of the bone microarchitectural changes caused by GIA in Nkx2-3^−/−^ and control BALB/c mice. Arthritic Nkx2-3^−/−^ (*n* = 2) and control BALB/c (*n* = 2) mice were anesthetized and micro-CT scans were made from the right hind limbs. Representative images show the dorsal (**A**) and (**B**) or plantar (**C**,**D**) or side views (**E**,**F**) of the arthritic legs from BALB/c (**A**,**C**,**E**) and Nkx2-3^−/−^ (**B**,**D**,**F**) mice, respectively. Pseudocolored images (**A**/**b**,**c**, **B**/**b**,**c**, **C**/**b**,**c**, **D**/**b**,**c**, **E**,**F**) show the bone densities (violet and blue colors indicate low density-; yellow, red and green colors indicate high-density areas, respectively). The white dashed line-surrounded rectangular areas indicated in **A**/**b**, **B**/**b**, **C**/**b** and **D**/**b** are shown with higher magnification in **A**/**c**, **B**/**c**, **C**/**c** and **D**/**d**.

Next, we wanted to visualize the radiological changes induced by GIA in Nkx2-3^−/−^ and control BALB/c mice. RA causes not only cartilage destruction but the adjacent bone is always affected as a result of the inflammation-induced osteoclast activity. Typical changes associated with RA are bone loss, osteophyte formation and, in the latter stages of the disease, ankylosis. So, we performed a micro-CT analysis of arthritic Nkx2-3^−/−^ and control BALB/c mice. As shown by the pseudocolor enhanced micro-CT images wild-type mice demonstrated marked osteophyte formation and bone surface irregularity predominantly in the tarsal and metatarsal region (Figure 2A,C,E). In comparison, Nkx2-3^−/−^ animals showed less severe detrimental bone structural damage secondary to the autoimmune arthritis manifested by a more limited surface porosity and periarticular inflammatory osteoporosis (Figure 2A,C,E).

**Figure A1:**
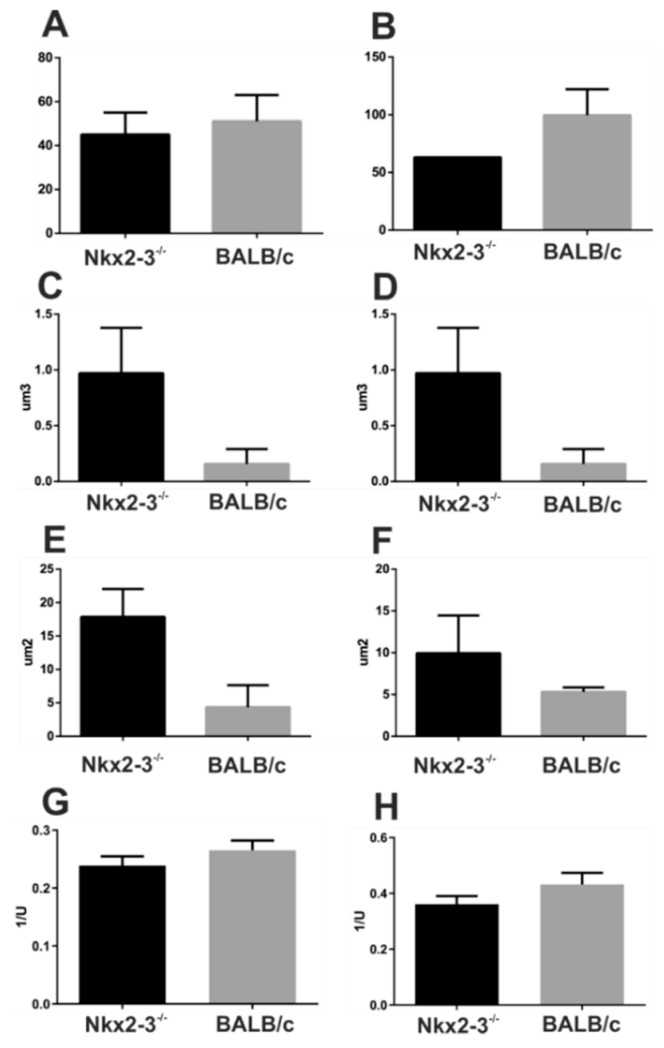
Comparison of the quantitative markers of bone microarchitecture in arthritic Nkx2-3^−/−^ and BALB/c mice based on the micro-CT scans (representative images are shown in Figure 2). We compared the bone pore number (Po.N) (**A**,**B**), volume (Po.V) (**C**,**D**) and surface (Po.S) (**E**,**F**), and the Bone Surface/Total Volume (BS/TV) values (**G**,**H**) of the metatarsal (**A**,**C**,**E**,**G**) and tarsal (**B**,**D**,**F**,**H**) regions from arthritic Nkx2-3^−/−^ (black bars) and BALB/c (gray bars) mice, respectively. Bars show the mean ± SEM values calculated from the data of 2 Nkx2-3^−/−^ and 2 BALB/c mice.

We also analyzed the micro-CT scans for some quantitative markers of the bone microarchitecture, e.g., number (Po.N), volume (Po.V) and surface of bone pores (Po.S), and the bone surface/Total Volume (BS/TV) values. In arthritic Nkx2-3 KO mice, the slightly decreased Po.N together with the increased Po.V and Po.S values could be due to decreased reactive bone formation, whereas the slightly decreased BS/TV value might indicate less osteophyte formation, all of which correlate with the milder arthritis (Figure A1).

### 2.3. Comparison of the G1-Specific Immune Response between Nkx2-3^−/−^ and BALB/c Mice

Given the lower severity and incidence of autoimmune arthritis in the Nkx2-3^−/−^ mice, next, we wanted to characterize the immune response against the G1 antigen and correlate it to the clinical picture. At the end of the experiments, mice were sacrificed and their spleens and sera were harvested for in vitro assays similarly to previous studies [5,27]. In the in vitro studies, we divided the Nkx2-3^−/−^ mice into arthritic and nonarthritic subgroups to see the possible differences in the immunological parameters.

**Figure 3:**
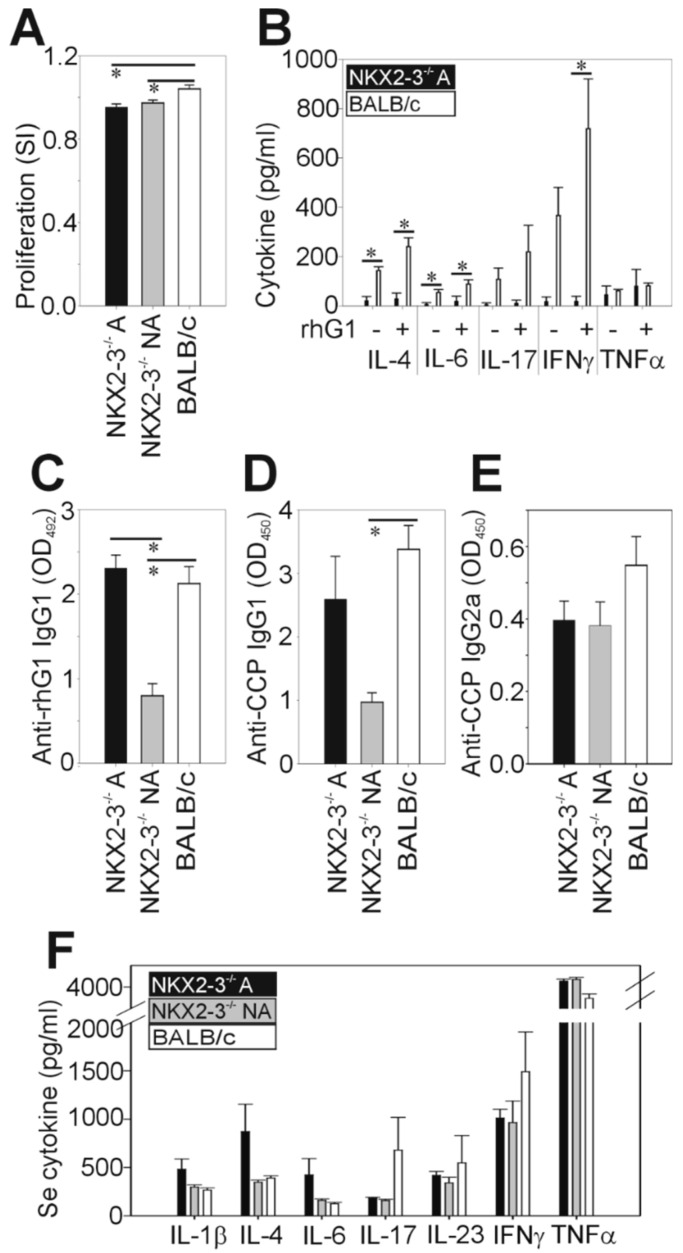
The comparison of the rhG1-induced immune response in Nkx2-3^−/−^ (*n* = 9) and control BALB/c (*n* = 9) mice. Nkx2-3^−/−^ mice were divided into arthritic (*n* = 5) and nonarthritic (*n* = 4) subgroups during the analysis. In all diagrams (**A**–**F**), bars show the mean ± SEM values calculated from *n* = 5 arthritic Nkx2-3^−/−^ mice (black), *n* = 4 nonarthritic Nkx2-3^−/−^ mice (gray) and *n* = 9 arthritic control BALB/c mice (white). Statistically significant differences (∗ *p* < 0.05) are indicated. (**A**) Proliferation of spleen cells was tested after incubation in the presence or absence of rhG1 for 5d in vitro. Bars represent the stimulation index (SI) calculated as a ratio of stimulated/nonstimulated values of the same mice. (**B**) In vitro cytokine production of spleen cells was tested after incubation in the presence or absence of rhG1 for 5 d. Cell culture supernatants were harvested and the specific cytokine concentrations were measured by sandwich ELISA. Note, spleen cells from the nonarthritic Nkx2-3^−/−^ group did not produce any measurable amount of cytokines. (**C**) The serum anti-rhG1-specific IgG1 antibodies were measured using indirect ELISA. Sera were diluted at 1:8000. Bars show the optical density values measured at 492 nm. (**D**,**E**) The serum anti-CCP-specific IgG1 and IgG2a antibodies were measured using indirect ELISA. Sera were not diluted. Bars show the optical density values measured at 450 nm. (**F**) Serum cytokine levels were measured with sandwich ELISA.

First, we characterized the antigen-induced proliferation of spleen cells. We found significantly decreased proliferation in both arthritic and nonarthritic Nkx2-3^−/−^ mice compared to the arthritic BALB/c mice (Figure 3A). Since Th1, Th2 and Th17 cytokines play a pivotal role in the regulation of GIA [8,28], next, we measured the cytokine production of the rhG1-stimulated spleen cell cultures (Figure 3B). We found that Nkx2-3^−/−^ spleen cells produced significantly less IL-4, IL-6 and IFNγ and markedly, but not significantly, less IL-17 than the corresponding BALB/c spleen cells (Figure 3B). There was no difference in the TNFα production of splenocytes isolated from Nkx2-3^−/−^ or control BALB/c mice (Figure 3B). Of note, nonarthritic Nkx2-3^−/−^ spleen cells produced no detectable amount of the tested cytokines even in the presence of rhG1.

Antibody production is an important laboratory parameter of GIA correlating with the severity of the disease [5]. So, after the characterization of the cellular immune response, we went on to measure the joint inflammation-related serum antibody levels of Nkx2-3^−/−^ and BALB/c mice. We found significantly decreased anti-rhG1 (Figure 3C) and anti-CCP (Figure 3D) IgG1 levels in the nonarthritic Nkx2-3^−/−^ mice sera compared to the arthritic BALB/c controls. The arthritic Nkx2-3^−/−^ mice had similar levels of anti-rhG1 (Figure 3C) and anti-CCP (Figure 3D) IgG1 to the arthritic BALB/c mice. Furthermore, the anti-CCP-IgG2a levels were lower (but not significantly) in both arthritic and nonarthritic Nkx2-3^−/−^ mice than in arthritic BALB/c controls (Figure 3E). In line with our expectations, we found that the sera of nonarthritic Nkx2-3^−/−^ mice contained lower levels of anti-rhG1 and anti-CCP IgG1 when compared to the arthritic Nkx2-3^−/−^ sera (Figure 3C,D). In contrast, there was no significant difference between the anti-CCP IgG2a levels of the sera from arthritic or nonarthritic Nkx2-3^−/−^ mice (Figure 3E).

Finally, we compared the serum pro- and anti-inflammatory cytokine concentrations of Nkx2-3^−/−^ and BALB/c mice. We found markedly, but not significantly, elevated serum levels of IL-1β, IL-4, IL-6 and TNFα and markedly decreased IL-17 and IFNγ in arthritic Nkx2-3^−/−^ mice when compared to the arthritic BALB/c controls (Figure 3F). In the case of IL-1β, IL-4 and IL-6, the serum concentrations were markedly, but not significantly, higher in the arthritic than in the nonarthritic Nkx2-3^−/−^ sera (Figure 3F).

### 2.4. Comparison of the Ca^2+^-Signaling in B and T Cells of Nkx2-3^−/−^ and BALB/c Mice

**Figure 4:**
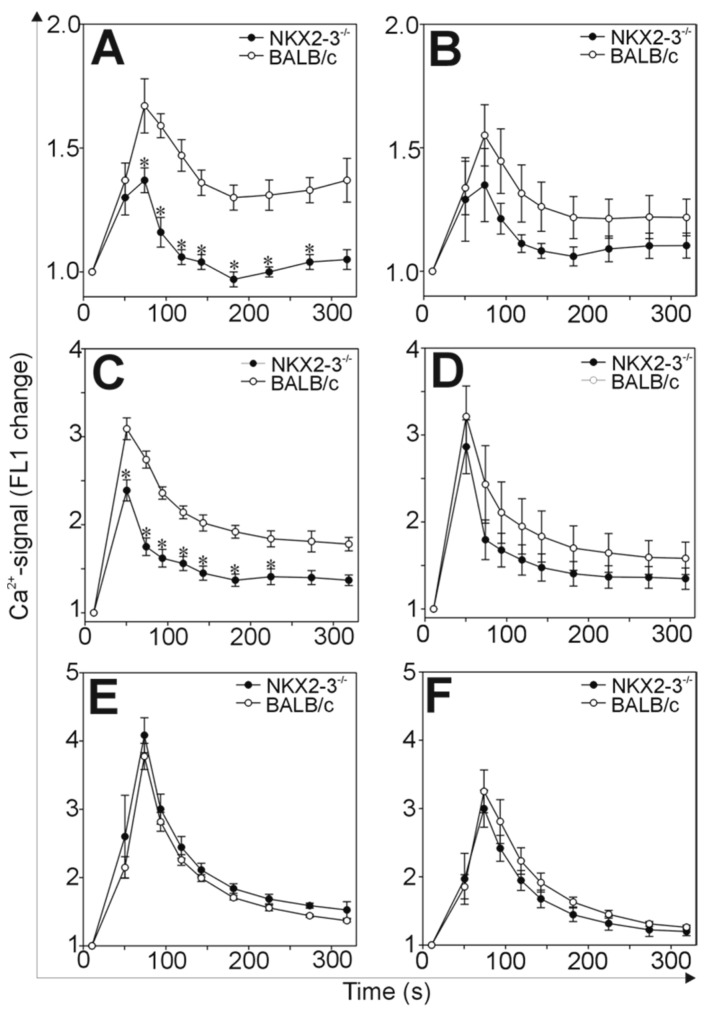
The comparison of the Ca^2+^-signals of B and T cells in Nkx2-3^−/−^ and control BALB/c mice. Cells were isolated from the inguinal (**A**,**C**,**E**) or mesenteric (**B**,**D**,**F**) lymph nodes of Nkx2-3^−/−^ and BALB/c mice and loaded with the Ca^2+^-specific indicator Fluo-3. Activation of B cells was induced by anti-IgM (**A**,**B**) or anti-IgG (**C**,**D**), activation of T cells was induced by anti-CD3 cross-linking (**E**,**F**). The changes in the intracellular Ca^2+^ levels of B or T cells were measured with a flow cytometer in the FL1 channel for five and a half minutes. Graphs show the time-dependent changes in the FL1 fluorescence (ratiometric with the intracellular Ca^2+^ level) as mean ± SEM values calculated from the data of *n* = 3 Nkx2-3^−/−^ and *n* = 3 BALB/c mice. * *p* < 0.05.

Since we found significant differences in the rhG1-induced immune responses of the Nkx2-3^−/−^ and BALB/c mice, we were curious about what could be in the background of such variations. In the case of GIA, similarly to RA, close cooperation between T and B cells is necessary for the development of autoimmunity [7]. Since the Nkx2-3 mutation affects mostly the B lymphocyte development and recirculation, we hypothesized that the activation of B cells might be impaired, which, in turn, led to ameliorated arthritis. To characterize the activation of the B cells, we isolated the inguinal and mesenteric lymph nodes from Nkx2-3^−/−^ or BALB/c control mice and loaded the lymphocytes with the Ca^2+^-specific indicator Fluo-3. Then, we activated the B cells with cross-linking of the BcR with anti-IgM or IgG antibodies and followed the intracellular Ca^2+^ signals (Figure 4).

We found significantly lower anti-IgM or anti-IgG-induced Ca^2+^-signal in the B cells isolated from the inguinal lymph nodes of Nkx2-3^−/−^ mice than those from BALB/c (Figure 4A,C). Similarly, B cells isolated from the mesenteric lymph nodes, showed decreased Ca^2+^-signal both after anti-IgM or anti-IgG-activation (Figure 4B,D) when compared to BALB/c controls, however, these differences did not reach statistical significance. Finally, although we were not expecting any changes in the T cell activation in Nkx2-3^−/−^ mice, we tested the Ca^2+^-signal of T cells after anti-CD3 cross-linking. As we expected, the Ca^2+^-signals of T cells isolated from both the inguinal and mesenteric lymph nodes were similar in Nkx2-3^−/−^ and BALB/c mice indicating that, indeed, the Nkx2-3 mutation caused an activation perturbance specifically in B cells.

## 3. Discussion

In the present study, we set out to study how Nkx2-3-deficiency impacted the development of autoimmune arthritis. To answer this question, we used a mouse RA model, GIA, whereby we immunized Nkx2-3^−/−^ and BALB/c control mice side-by-side with the rhG1 antigen. While the spleen plays important roles in the T-dependent immune responses [11,29], and in GIA it also serves as an activation niche for autoreactive lymphocytes [8,30], in Nkx2-3^−/−^ mice, with severely damaged spleen, GIA could still be induced. This result shows that in GIA the spleen’s role is not exclusive in the activation of autoreactive lymphocytes, and confirms that the lymph nodes and perhaps other lymphatic or extralymphatic tissues [8] may play an equally important role in the disease induction. On the other hand, we observed lower incidence and decreased arthritis severity in the Nkx2-3^−/−^ mice compared to the wild-type controls, thus, the splenic defects had a significant impact on the rhG1-induced immune reaction. During the induction of (P)GIA, we immunized the mice with PG extracts/rhG1 and DDA intraperitoneally which led to the local activation of T lymphocytes both in the peritoneal cavity and the mesenteric lymph nodes followed by the systemic immune response in which the spleen is involved [8]. The local activation of Th1 and Th17 cells in the peritoneal cavity is of special importance because it was a specific feature of BALB/c mice which are the only susceptible mouse strain for (P)GIA [8]. The present results in Nkx2-3^−/−^ mice might also support this: the local peritoneal activation could remain unchanged, however, the systemic response, developing in the spleen is missing, which could be responsible for the weaker arthritis.

As it was previously described there is a significant B cell trafficking between the peritoneal cavity and the spleen [26]. Although there was a preferential homing of the peritoneal B1 and B2 cells towards other serosa surfaces like the pleura, however, some B1 and B2 cells migrated from the peritoneal cavity into the spleen [26]. Since in Nkx2-3^−/−^ mice, the spleen microarchitecture is seriously defective, with abnormal adhesion molecule expression and vessel formation [29] most likely, the above-mentioned B cell trafficking is defective [29], in addition to the absence of MZ B cells involved in antigen delivery for GC initiation [21]. B lymphocytes not only play a role as precursors for antibody-producing plasma cells in autoimmune arthritis but have an equally important role as antigen-presenting cells [31]. Based on the above-mentioned works [26,29], we propose that the local, peritoneal antigen presentation by B cells could remain unchanged, however, due to the potential lack of splenic homing (and the severely reduced GC formation in the spleen upon T-dependent antigen challenge [21]), the systemic activation could be impaired. The subsequently weaker immune response is mirrored in the decreased splenic proliferation and cytokine production. This was particularly pronounced in the case of lymphocyte-derived cytokines (IL-4, IL-6, IL-17 and IFNγ), but, in the case of TNFα, we measured approximately equal amounts in Nkx2-3^−/−^ and control BALB/c spleen cell cultures, showing that the macrophages were not affected.

In humans, the potential pathogenic role of the Nkx2-3 transcription factor was found in inflammatory bowel diseases (Crohn’s and ulcerative colitis) so far [23,24] and also suggested in spondylarthritis more recently [32]. In mice, the absence of Nkx2-3 proved to be protective in DSS-induced colitis through an IL-22-independent mechanism [18]. To our knowledge, this was the first experiment where the Nkx2-3^−/−^ mutation was investigated in the context of autoimmune arthritis. Based on the data presented in this study, Nkx2-3 is not only involved in intestinal inflammatory diseases, but also affects autoimmune arthritis.

An important finding of the present study was that not all Nkx2-3^−/−^ mice developed arthritis upon rhG1 immunization: in all experiments, 20–40% of Nkx2-3^−/−^ mice remained healthy contrary to BALB/c mice (>90% incidence, as seen here and in previous studies [5,27]). To decipher what could be the reason why certain immunized mice did not develop arthritis, we analyzed their immune response parameters separately. We measured considerably lower anti-rhG1 and anti-CCP IgG1 antibody levels in the sera of nonarthritic than in the sera of arthritic Nkx2-3^−/−^ mice, respectively. This alone could explain the differences in the arthritis, since it has been shown in several earlier studies that the serum antibody levels against the proteoglycan aggrecan (and its immunodominant region: G1 domain) and CCP show the strongest correlation with the severity of GIA [33,34]. Furthermore, in nonarthritic Nkx2-3^−/−^ mice, the serum concentrations of IL-1β, IL-4 and IL-6 were lower than in arthritic Nkx2-3^−/−^ mice. We hypothesize that in those Nkx2-3^−/−^ mice, which did not develop arthritis, the B cell activation and/or antigen presentation was inadequate to induce sufficient antibody production and autoreactive T cell activation. However, further and more detailed investigation would be needed to adequately answer this question.

Even in those Nkx2-3^−/−^ mice which did develop GIA, there were significantly milder symptoms (lower clinical scores, lesser edema, radiologically decreased cartilage and bone destruction). This was in line with those serum parameters which were clearly different from the control BALB/c mice. Specifically, although the anti-rhG1 and anti-CCP-IgG1 antibody levels were similar in the arthritic Nkx2-3^−/−^ mice and the controls, the anti-CCP-IgG2a antibodies were produced in lesser amounts. Additionally, the concentrations of signature cytokines of GIA [5], and likewise, RA [2], IFNγ and IL-17 were markedly lower, whereas the concentrations of IL-1β, IL-4 and IL-6 were markedly higher in Nkx2-3^−/−^ than in BALB/c mice. Overall, these markers suggest a stronger Th2 activation (primarily indicated by IL-4 and anti-CCP IgG2a) in Nkx2-3^−/−^ mice instead of the characteristic Th1/Th17 dominated immune response seen in GIA of BALB/c mice [5,8,28]. Since Th2 cytokines have primarily anti-inflammatory effects [11] and have been shown to ameliorate PGIA [35], we suggest that this slight shift towards Th2 measured in Nkx2-3^−/−^ mice might explain the milder arthritis.

As described here, and earlier [5], similarly to RA, in GIA mice significant anti-CCP antibody production can be detected. The process of citrullination and the role of autoantibodies against these modified antigens is of special interest in RA. ACPA production is promoted by environmental factors like smoking and genetic predispositions such as *HLA-DRB1* [36]. Several studies reported that ACPA positive patients are prone to have more joint erosions [36,37]. ACPA form immune complexes with the citrullinated peptides leading to the activation of macrophages and proinflammatory cytokine production, as well as osteoclastogenesis [36,37]. ACPA is highly specific to RA and can be early detected, even before the onset of RA. Therefore, it is used as a specific diagnostic marker for RA [38]. Recently, it was also found that the pathogenic ACPA are hyperglycosylated which might be regulated by Th17 cells [37]. Since in GIA Th17 activation was observed it is tempting to speculate that altered glycosylation might also occur which could contribute to the pathologic immune reaction against the cartilage antigen components.

Finally, to find a cellular mechanism in the background of the above-detailed immune response differences, we turned our attention to the activation of B cells. B cell activation starts with the engagement of the BcR by the antigen and followed by a well-characterized line of biochemical events including the phosphorylation of cytoplasmic signaling proteins and the transient elevation of the cytoplasmic Ca^2+^-level [11]. We studied the latter and found that in mesenteric and inguinal lymph node B cells of Nkx2-3^−/−^ mice, in vitro stimulation with both anti-mouse IgM and anti-mouse IgG caused a weaker Ca^2+^-signal compared to BALB/c controls. At the same time, the Ca^2+^-signal in Nkx2-3^−/−^ T cells remained unchanged, showing that this was a B-cell-specific alteration. This decreased B cell activation capacity of Nkx2-3^−/−^ mice was also seen on phospho-blots from some preliminary experiments (data not shown), however, further analysis is needed to clarify which protein(s) could be involved in the signaling changes. These data are in harmony with those described earlier: increased expression of Nkx2-3 in B cells led to Syk and Lyn phosphorylation, elevated basal Ca^2+^-level and anti-IgM-induced Ca^2+^-signal [25], the exact opposite to what we found here, in the absence of Nkx2-3. The decreased antigen-driven B cell activation could lead to weaker B cell proliferation and differentiation which, in turn, could explain the less pronounced immune response and the consequently milder autoimmune arthritis.

In conclusion, the complex immune response changes in Nkx2-3^−/−^ mice due to the defective spleen structure and function could explain the reduced severity and lower incidence of autoimmune arthritis. At the cellular level, we found weaker B cell activation which might, at least in part, be responsible for the altered immune response. These data add to our knowledge about the significance of the spleen in the development of autoimmunity, and hopefully serve as a starting point for future studies.

## 4. Materials and Methods

### 4.1. Mice

We used 4–5 months old [39] female Nkx2-3-deficient (Nkx2-3^−/−^) [29] and BALB/c mice. Animals were kept and bred in the transgenic mouse facility of the Department of Immunology and Biotechnology under conventional conditions at 24 ± 2 °C with a controlled 12/12 h light/dark cycle. Mice used in experiments were housed in groups of five and they received acidified water and food ad libitum.

All animal experiments were performed in accordance with the regulations set out by the Animal Welfare Committee of the University of Pécs (BA02/2000-48/2017 (06/27/2017)).

### 4.2. Induction and Assessment of Recombinant Human G1-Induced Arthritis

To induce arthritis, Nkx2-3^−/−^ and BALB/c mice were immunized side-by-side as described previously [5]. Briefly, mice received intraperitoneal injections of 40 µg rhG1 antigen mixed with dimethyl-dioctadecyl-ammonium (DDA) adjuvant dissolved in PBS, on Days 0, 21 and 42. The clinical signs of arthritis were monitored regularly after the second immunization: all mice were examined every second day and the symptoms were quantified using a clinical scoring system ranging from 0 to 4 based on the swelling, redness and ankylosis of the joints of the paws (maximum possible score 16) as described before [4,5]. The diameters of the inflamed front and hind paws and ankles were measured using digital calipers with an accuracy of 0.01 mm two weeks after the third immunization. Three weeks after the last immunization, mice were sacrificed, then blood sera, spleens and lymph nodes were collected for further in vitro studies.

### 4.3. Micro-CT

The right hind paws of mice were scanned under anesthesia with i.p. ketamine (120 mg/kg; Calypsol, Gedeon Richter, Budapest, Hungary) and xylazine (6 mg/kg; Sedaxylan, Eurovet Animal Health, Bladel, The Netherlands) using a SkyScan 1176 in vivo micro-CT system (Bruker, Kontich, Belgium). A 0.5 mm Al filter was used, the voxel size was 17.5 μm, tube voltage was 50 kV, tube current was set to 500 μA. 3D reconstructions of the scans were made with the CT Analyzer software, and representative pseudocolor images were generated to highlight bone erosions and osteophyte formation.

### 4.4. In Vitro Spleen Cell Culture

Spleens were isolated and homogenized mechanically, then the spleen cells were cultured in DMEM supplemented with 10% fetal calf serum on 48-well plates (1.8 × 10^6^ cells in 600 μL medium/well), in the presence or absence of 1.5 µg rhG1 antigen for 5 d. Supernatants were collected and stored in −20 °C and later used for ELISA measurements.

### 4.5. Antigen-Specific Proliferation Assay

Another part of the spleen cells were cultured in DMEM supplemented with 10% fetal calf serum, in the presence or absence of 1.5 µg rhG1 antigen in triplicates on 96-well plates (3 × 10^5^ cells in 200 μL medium/well) for 5 d. Promega CellTiter^96®^ Nonradioactive Cell Proliferation Assay (Promega, Madison, WI, USA) was used to measure the proliferation rate according to the manufacturer’s instructions.

### 4.6. ELISA Measurements

The specific cytokine concentrations (IL-1β, IL-4, IL-6, IL-17, IL-23, IFN-γ and TNF-α) were measured in the blood sera and the supernatants of in vitro-cultured spleen cells using sandwich ELISA (R&D Systems, Minneapolis, MN, USA), according to the manufacturer’s instructions.

The serum-concentration of rhG1 antigen-specific antibodies was measured using indirect ELISA as described earlier [27]. Briefly, 96-well ELISA plates were coated overnight at room temperature with the rhG1 antigen (0.1 μg/well in 100 μL carbonate coating buffer). After overnight incubation, plates were incubated for 1 h with 1.5% nonfat dry milk (NFDM) in PBS blocking buffer, followed by washing 5 times with 0.5% Tween in PBS. Next, diluted sera were added to the plate and incubated for 2 h at room temperature, then washed 5 times with 0.5% Tween in PBS. After that, anti-IgG1-peroxidase (BD Bioscience, San Jose, CA, USA) secondary antibody was added to the plate and incubated for 2 h at room temperature. The results were detected using orthophenylenediamine chromophore and H_2_O_2_ substrate.

The anti-CCP IgG1 and IgG2a antibody levels of sera were determined using the commercially available Immunoscan CCP Plus ELISA kit (SVAR, Malmö, Sweden) with slight modification. For the development of the reactions, we used anti-mouse-IgG1-peroxidase or anti-mouse-IgG2a-peroxidase (both from BD Bioscience, San Jose, CA, USA) secondary antibody instead of the kit’s secondary reagent.

### 4.7. Ca^2+^ Signaling Measurements

The intracellular Ca^2+^ levels were measured using a flow cytometer with the Fluo-3 indicator as described before [40,41]. Briefly, single-cell suspensions were prepared from the lymph nodes of the arthritic mice and suspended in RPMI supplemented with 5% FBS and 2M CaCl_2_ (1 × 10^6^ cells/mL). Cells were loaded with Fluo-3-AM (Invitrogen, Carlsbad, CA, USA) for 30 min at 37 °C in humidified air with 5% CO_2_. Using a flow cytometer, we gated on the lymphocytes based on FSC/SSC parameter distribution. The Fluo-3 fluorescence which is proportional to the intracellular Ca^2+^ level [40] was detected in the FL1 channel. The baseline Fluo-3 fluorescence was measured for 1 min, then cells were stimulated with anti-mouse-IgM, IgG, or anti-mouse CD3 followed by secondary anti-hamster antibodies, measurements for five and a half minutes. Data were analyzed by the Cell Quest software (BD Biosciences, San Jose, CA, USA). The FL1 mean fluorescence intensity values were calculated along the time axis of the plots, and these were divided by the baseline fluorescence value, thereby the Ca^2+^ signal was expressed as FL1 change [41]. Please note that all Ca^2+^ measurements were performed using unsorted lymph node cells and the B or T cell activation was only distinguished based on the specific nature of the activation: anti-mouse-IgM or IgG activates only B cells through the BcR, whereas anti-CD3 cross-linking activates T cells selectively.

### 4.8. Statistical Analysis

All values are presented as mean ± standard error of mean (SEM). Student’s *t*-test was used to compare the experimental groups. *p*-values ˂ 0.05 were considered statistically significant.

## Abbreviations

a-CCP	Anticyclic citrullinated peptide antibody
APCs	Antigen-presenting cells
Ca^2+^	Calcium
CD	cluster of differentiation
DDA	Dimethyl-Dioctadecyl-ammonium adjuvant
DMEM	Dulbecco’s modified Eagle’s medium
ELISA	Enzyme-linked immunosorbent assay
FCS	Fetal calf serum
FSC	Forward scatter
GC	Germinal center
GIA	Recombinant human G1-induced arthritis
i.p	Intraperitoneal
IFN-γ	Interferon γ
IGH	Immunoglobulin heavy chain gene
IL	Interleukin
MADCAM-1	Mucosal vascular addressin cell adhesion molecule 1
MZ	Marginal zone
NFDM	Nonfat dry milk
NF-kB	Nuclear factor kappa-light-chain-enhancer of activated B cells
Nkx2-3	Nirenberg-Kim (NK) 2 homeobox 3
Nkx2-3^−/−^	Nkx2-3-deficient mouse (homozygous)
PBS	Phosphate buffer solution
PI3K	Phosphoinositide 3-kinases
PKB	Protein Kinase B
PTPN2	Tyrosine-protein phosphatase nonreceptor type 2
RA	Rheumatoid arthritis
rhG1	Recombinant human G1
RPMI	Roswell Park Memorial Institute Medium
SSC	Side scatter
TF	Transcription factor
Th	T helper cell
TNF-α	Tumor necrosis factor α
VEGF	Vascular endothelial growth factor
